# Proficiency of clinical laboratories in and near Monterrey, Mexico, to detect vancomycin-resistant enterococci.

**DOI:** 10.3201/eid0501.990117

**Published:** 1999

**Authors:** L C McDonald, L R Garza, W R Jarvis

**Affiliations:** Centers for Disease Control and Prevention, Atlanta, Georgia 30333, USA.

## Abstract

Early detection of vancomycin-resistant enterococci is important for preventing its spread among hospitalized patients. We surveyed the ability of eight hospital laboratories in and near Monterrey, Mexico, to detect vancomycin resistance in Enterococcus spp. and found that although laboratories can reliably detect high-level vancomycin resistance, many have difficulty detecting low-level resistance.


### Proficiency of Clinical Laboratories in and near Monterrey, Mexico, To Detect Vancomycin-Resistant Enterococci

**L. Clifford McDonald,* Luis R. Garza,† and William R. Jarvis***
*Centers for Disease Control and Prevention, Atlanta, Georgia, USA; and
†Hospital Jose A. Muguerza, S.A. De C.V., Monterrey Nuevo León, Mexico

Early detection of vancomycin-resistant enterococci is important for preventing its spread among hospitalized patients. We surveyed the ability of eight hospital laboratories in and near Monterrey, Mexico, to detect vancomycin resistance in *Enterococcus* spp. and found that although laboratories can reliably detect high-level vancomycin resistance, many have difficulty detecting low-level resistance.

Since vancomycin-resistant enterococci (VRE) were first reported in the late 1980s, their geographic distribution and importance as nosocomial pathogens have continued to increase worldwide. In the United States, the percentage of states with a National Nosocomial Infections Surveillance System hospital reporting one or more patients with VRE infection increased from 27% (1989–1993) to 44% (1994–1995) (1). Among enterococci causing infection in these hospitals, the percentage resistant to vancomycin increased from 0.4% (1989) to 10.8% (1995) in intensive care unit patients and from 0.3% (1989) to 10.4% (1995) in nonintensive care unit patients (1).

Although early detection of VRE is important for preventing its spread among hospitalized patients, clinical laboratories in the United States have difficulty detecting VRE, especially those with intermediate or low-level resistance, characteristic of VRE with the VanB phenotype (2,3). Certain automated and manual antimicrobial susceptibility test systems are associated with the inability of laboratories to detect VRE. Laboratories outside the United States (e.g., in Argentina [4]) may have difficulty detecting VRE for largely the same reasons: limitations of susceptibility test systems. In addition, laboratories outside the United States, Canada, and Europe may face language and financial barriers to accessing information important for updating their methods and optimizing VRE detection.

Address for correspondence: William R. Jarvis, Hospital Infections Program, Centers for Disease Control and Prevention, 1600 Clifton Road, Mail Stop E69, Atlanta, GA 30333, USA; fax: 404-639-6459; e-mail:wrj1@cdc.gov.

No VRE have been reported from hospitals in Mexico. However, because VRE have been reported from hospitals in Texas, and VanB has been described as the predominant phenotype in at least one hospital in the Houston area (5), we assessed the ability of clinical laboratories in and near Monterrey (in northeastern Mexico) to detect VRE.

### Study Protocol

The laboratory survey and data collection were performed in July-August 1997. Five strains of enterococci (two *Enterococcus faecium*, two *E. faecalis*, one *E. gallinarum*), with or without resistance to vancomycin, were coded as isolates 1 through 5 (Table). Four of these had been used in proficiency surveys in the United States (3) and Argentina (4). The fifth isolate came from the American Type Culture Collection (ATCC 29212). The isolates were distributed,

Table. Characteristics of enterococcal study isolates and results of enterococcal susceptibility testing, by category

| Isolate no. and species | MIC (µg/ml) | Vancomycin phenotype | No. of labs[a] (n = 8) S | I | R |
|---|---|---|---|---|---|
| 1. *E. faecium* | 512 | VanA | 0 | 0 | 8[b] |
| 2. *E. faecium* | 64 | VanB-like | 2 | 1 | 5[b] |
| 3. *E. faecalis* | 16-32 | VanB | 2 | 1[b] | 5[b] |
| 4. *E. gallinarum* | 8 | VanC | 1 | 5[b] | 2 |
| 5. *E. faecalis* | ≤4 | Susceptible | 7[b] | 1 | 0 |

[a]Laboratories reporting susceptibility to vancomycin; S = susceptible, I = intermediate, R = resistant.
[b]Correctly identified.

along with standardized susceptibility test results forms, to eight clinical laboratories (seven within and one near Monterrey).

Laboratories were blinded to the susceptibility patterns of the isolates and asked to test the five isolates for resistance to vancomycin with the antimicrobial susceptibility testing method they routinely used. The laboratorians recorded disk zone sizes or MICs and their interpretation of the results (susceptible, intermediate, or resistant), in addition to species identification methods used, zone size breakpoints used for disk diffusion, existence of an antimicrobial control program, and hospital demographic characteristics. No information was collected regarding the version of software program used with automated susceptibility test systems. The Centers for Disease Control and Prevention (CDC) analyzed the forms and classified errors in vancomycin susceptibility testing as very major (reporting a resistant strain as susceptible), major (reporting a susceptible strain as resistant), minor (reporting an intermediate or resistant strain as susceptible or intermediate, respectively), or very minor (reporting a susceptible or intermediate strain as intermediate or resistant, respectively).

MICs were determined by broth microdilution and disk diffusion testing (3). In addition, a polymerase chain reaction assay was used to confirm the presence of the *vanA* resistance determinant in organism 1 (3).

### Study Findings

The eight participating laboratories each serviced one hospital with a median bed count of 148 (70 to 185). All but one hospital had neonatal, pediatric, and adult intensive care units; half were teaching hospitals. Only two laboratories reported an antimicrobial use control program in place in their hospital.

The antimicrobial susceptibility testing methods used were the Sceptor system (Becton-Dickinson Microbiology Systems, Cockeysville, MD) (three laboratories); Vitek (bioMerieux, St. Louis, MO) (two); standard disk diffusion (two); and Microscan Autoscan (Dade International, West Sacramento, CA) (one). Of the two laboratories that used disk diffusion, one used outdated breakpoint zone sizes; the other used breakpoints currently recommended by the National Committee for Clinical Laboratory Standards (NCCLS).

### Vancomycin Resistance Detection

All laboratories correctly detected the high-level vancomycin resistance in isolate 1 (high-level vancomycin resistance [MIC 512 µg/ml] of the VanA phenotype) (Table). Only five laboratories reliably detected the low-level resistance (MIC 64 µg/ml) typical of the VanB-like phenotype possessed by isolate 2; laboratories made two very major errors reporting this resistant isolate as susceptible. Six laboratories correctly categorized isolate 3 with the VanB phenotype (MIC 16-32 µg/ml) as intermediate or resistant; two laboratories committed minor errors by reporting this isolate as susceptible. Five laboratories correctly categorized isolate 4; one laboratory committed a minor error, and two laboratories committed very minor errors. Of the eight laboratories, seven correctly identified vancomycin susceptibility in isolate 5.

Of the 15 test results from the three hospitals using the Sceptor system, 3 (20%) were errors (all minor). Of the 10 results from the two hospitals using disk diffusion, four (40%) were errors. The laboratory using outdated NCCLS zone-size breakpoints committed one very major and one very minor error; the laboratory using the current NCCLS breakpoints committed two very minor errors. The two hospitals using the Vitek automated system generated 10 vancomycin susceptibility test results; two were errors (one very major and one minor).

### Species Identification

Of the participating laboratories, three used the Sceptor system to identify species, two used the Vitek system, one used Microscan Autoscan, one used the Pasco system (Difco, Wheatridge, CO), and one did not report its identification method. Seven laboratories correctly identified isolates 1 and 2, both *E. faecium*; five laboratories correctly identified isolate 3, an *E. faecalis;* none correctly identified isolate 4, an *E. gallinarum* (six laboratories identified it as *E. faecalis* and two as *E. faecium*); and all laboratories correctly identified isolate number 5, an *E. faecalis*.

### Conclusions

Because VRE may be transmitted easily by health-care workers from infected to uninfected patients, who may become colonized and serve as reservoirs for transmission, the delay caused by failure of the laboratory to detect VRE from an infected patient may facilitate the emergence of VRE in a hospital or a group of hospitals in a geographic region. Early studies suggested laboratories were more likely to miss intermediate- or low-level vancomycin resistance in enterococci if they relied on commercial automated test systems (3,6). More recent studies suggest persistent problems with the Vitek instrument (2). In addition, disk diffusion is not sufficiently sensitive in detecting low- and intermediate-level resistance to vancomycin when used as the sole method (2,4). Despite revision of NCCLS-recommended breakpoint zone sizes (7), disk diffusion remains relatively insensitive, possibly because susceptible and resistant zone sizes cluster around breakpoints. In addition, laboratory personnel may not consistently follow recommendations to read plates with transmitted light after a full 24 hours' incubation (7). Microscan has shown improved performance (2), likely due to software and hardware revisions (6).

Of the 40 test results in our study, 9 (23%) were erroneous compared with results at CDC; 2 (5%) were very major errors. In comparison, of 335 vancomycin susceptibility test results in 67 New Jersey laboratories, 111 (33%) were erroneous, with 20 (6%) very major errors (3). Of 25 vancomycin susceptibility test results in four Argentine laboratories, 11 (44%) were erroneous, with 2 (8%) very major errors (4). Although our results compared favorably with those of the New Jersey and Argentina studies, which used four test strains we used, the rate of very major errors is unacceptably high, especially for low-level resistance.

Some errors may have been caused by inadequately skilled personnel. One laboratory was using outdated zone-size breakpoints, despite 1992 revisions, a reminder that instituting contemporary methods in some laboratories may be difficult and lead to incorrect epidemiologic data. However, the overall lower error rate compared with previous studies and the association of errors with certain test methods suggest that limitations of test methods were primarily responsible for inaccuracies. Too few vancomycin susceptibility tests were performed to ascertain relative performance of test methods. However, the 20% error rate and lack of major errors obtained by the Sceptor system, compared with higher error rates and very major errors from laboratories that used the Vitek and disk diffusion methods, are consistent with previous results (8).

NCCLS recommends the use of an agar screen plate consisting of brain heart infusion agar containing 6 μg/ml of vancomycin to detect low- and intermediate-level vancomycin resistance (9). We recommend that laboratories in the Monterrey area use a supplemental brain heart infusion screening agar to test for vancomycin resistance in enterococci isolated from selected clinical specimens (e.g., blood, urine, sterile body sites). Because screening agar has a sensitivity of 100% and specificity of 96% to 99% (2), we recommend that isolates that grow on the screening agar be reported as resistant unless repeat testing with a reference MIC method suggests otherwise.

Some enterococci (e.g., *E. gallinarum* and *E. caseliflavus*), which have intermediate-level vancomycin resistance known as the VanC phenotype, rarely cause human infection. Isolation of this form of VRE, therefore, does not have the same public health importance as that of vancomycin-resistant *E. faecium* or *E. faecalis*. Therefore, to focus efforts on controlling antimicrobial resistance in species likely to cause serious human infection, laboratories must correctly identify VRE to the species level. Both *E. gallinarum* and *E. casseliflavus* can be differentiated from other enterococci on the basis of their motility. *E. casseliflavus* may be easily differentiated from *E. faecium* or *E. faecalis* by its yellow pigment; in contrast, *E. gallinarum* may not be reliably identified unless a motility test is performed with the appropriate media (10). A new conventional biochemical test for methyl-α-D-glycopyranoside (MDG) may be even more reliable than motility in differentiating *E. gallinarum* from *E. faecium* and *E. faecalis* (11).

The laboratories' difficulty in detecting low- and intermediate-level vancomycin resistance and in correctly identifying enterococci to the species level, especially *E. gallinarum,* suggests a need for additional tests to aid in the early detection and correct identification of VRE. Although no VRE have been reported from Mexico, the study area is close to areas in the United States where VRE have been reported and the VanB phenotype may be predominant (5).

Dr. McDonald is an associate investigator in the division of Clinical Research, National Health Research Institute, Taipei, Taiwan. He is assisting with the establishment of the Microbial Infections Reference Laboratory to coordinate surveillance and conduct research in antimicrobial resistance in Taiwan and elsewhere in Southeast Asia. His research interests include the epidemiology of antimicrobial resistance and methods to control its spread.

### References

1. Gaynes R, Edwards J, the NNIS system. Nosocomial vancomycin-resistant enterococci in the United States, 1989-1995: the first 1000 isolates [abstract]. Infect Control Hosp Epidemiol 1996;17:P18.
2. Rosenberg J, Tenover FC, Wong J, Jarvis W, Vugia DJ. Are clinical laboratories in California accurately reporting vancomycin-resistant enterococci? J Clin Microbiol 1997;35:2526-30.
3. Tenover FC, Tokars J, Swenson J, Paul S, Spitalny K, Jarvis W. Ability of clinical laboratories to detect antimicrobial agent-resistant enterococci. J Clin Microbiol 1993;31:1695-9.
4. Cookson ST, Lopardo H, Marin M, Arduino R, Rial MJ, Altschuler M, et al. Study to determine the ability of clinical laboratories to detect antimicrobial-resistant *Enterococcus* spp. in Buenos Aires, Argentina. Diagn Microbiol Infect Dis 1997;29:107-9.
5. Coque TM, Tomayko JF, Ricke SC, Okhyusen PC, Murray BE. Vancomycin-resistant enterococci from nosocomial, community, and animal sources in the United States. Antimicrob Agents Chemother 1996;40:2605-9.
6. Iwen PC, Kelly DM, Lindner J, Hinrichs SH. Revised approach for identification and detection of ampicillin and vancomycin resistance in Enterococcus species by using MicroScan panels. J Clin Microbiol 1996;34:1779-83.
7. Swenson JC, Ferraro MJ, Sahm DF, Charache P, the National Committee for Clinical Laboratory Standards Working Group on Enterococci, Tenover FC. New vancomycin disk diffusion breakpoints for enterococci. J Clin Microbiol 1992;30:2525-8.
8. Tenover FC, Swenson JM, O'Hara CM, Stocker SA. Ability of commercial and reference antimicrobial susceptibility testing methods to detect vancomycin resistance in enterococci. J Clin Microbiol 1995;33:1524-7.
9. National Committee for Clinical Laboratory Standards. Methods for dilution antimicrobial susceptibility tests for bacteria that grow aerobically. 4th ed. Approved Standard. M7-A4. Villanova (PA): The committee; 1997.
10. Facklam RR, Sahm DF. Enterococcus. In: Murray PR, editor. Manual of Clinical Microbiology. 6th ed. Washington: American Society for Microbiology; 1995. p. 308-14.
11. Turenne CY, Hoban DJ, Karlowsky JA, Shanel GG, Kabani AM. Screening of stool samples for identification of vancomycin-resistant enterococcus isolates should include the methyl-α-D-glycopyranoside test to differentiate nonmotile *Enterococcus gallinarum* from *E. faecium*. J Clin Microbiol 1998;36:2333-5.

